# Left Ventricular global longitudinal strain predicts heart failure readmission in acute decompensated heart failure

**DOI:** 10.1186/s12947-017-0098-3

**Published:** 2017-03-15

**Authors:** Simone Romano, Ibrahim N. Mansour, Mayank Kansal, Hana Gheith, Zachary Dowdy, Carolyn A. Dickens, Cassandra Buto-Colletti, June M. Chae, Hussam H. Saleh, Thomas D. Stamos

**Affiliations:** 1 Division of Cardiology, Department of Medicine, University of Illinois at Chicago, 840 S. Wood Street, M/C 715, Chicago, IL 60612 USA; 2 Department of Medicine, University of Verona, Ospedale policlinico Borgo Roma, Piazzale scuro 10, 37134 Verona, Italy

**Keywords:** Heart failure, Echocardiography, Strain analysis

## Abstract

**Background:**

The goal of this study was to determine if left ventricular (LV) global longitudinal strain (GLS) predicts heart failure (HF) readmission in patients with acute decompensated heart failure.

**Methods and results:**

Two hundred ninety one patients were enrolled at the time of admission for acute decompensated heart failure between January 2011 and September 2013. Left ventricle global longitudinal strain (LV GLS) by velocity vector imaging averaged from 2, 3 and 4-chamber views could be assessed in 204 out of 291 (70%) patients. Mean age was 63.8 ± 15.2 years, 42% of the patients were males and 78% were African American or Hispanic. Patients were followed until the first HF hospital readmission up to 44 months. Patients were grouped into quartiles on the basis of LV GLS. Kaplan-Meier curves showed significantly higher readmission rates in patients with worse LV GLS (log-rank *p* < 0.001). After adjusting for age, sex, history of ischemic heart disease, dementia, New York Heart Association class, LV ejection fraction, use of angiotensin converting enzyme inhibitors or angiotensin receptor blockers, systolic and diastolic blood pressure on admission and sodium level on admission, worse LV GLS was the strongest predictor of recurrent HF readmission (*p* < 0.001). The ejection fraction was predictive of readmission in univariate, but not in multivariate analysis.

**Conclusion:**

LV GLS is an independent predictor of HF readmission after acute decompensated heart failure with a higher risk of readmission in case of progressive worsening of LV GLS, independent of the ejection fraction.

## Background

Left ventricular ejection fraction (LVEF) is the most commonly used parameter of systolic function [1]. It is essential for the management of heart failure (HF) patients, particularly to guide therapy and for prognostication [2, 3]. Recently there has been great interest in development of novel quantitative methods to assess systolic function [4, 5]. One promising technique is two-dimensional speckle tracking which can provide information on the rate of segmental and global myocardial deformation. Global Longitudinal Strain (GLS) is defined as the change of length of a tissue normalized to its original length ([L-L_0_]/L_0_).

Prior studies have reported the usefulness of GLS for prognostic stratification of HF outpatients [6–9], however only one previous study found this parameter to be predictive in patients admitted to the hospital with acute decompensated heart failure (ADHF) in a mostly white European patient population (98%) [10].

We hypothesized that GLS could be a useful predictor of readmission in a mostly African American patient population hospitalized with ADHF.

## Methods

This was a single-center retrospective observational study that involved chart and medical record reviews, and analysis of de-identified clinical data and previously recorded echocardiographic tracings. Chart review was performed on 291 patients, aged ≥18 years, who were admitted to the University of Illinois Hospital and Health Sciences System (UI-Health) with the primary admission diagnosis of ADHF from January 2011 thru September 2013.

Inclusion criteria consisted of patients aged 18 years or older who had been admitted between January 2011 and September 2013 at the University of Illinois Hospital and Health Sciences System with the diagnosis of heart failure, either as new diagnosis or as acute decompensation of chronic HF, had a complete Transthoracic Echocardiography, and did not meet any of the exclusion criteria.

Exclusion criteria consisted of age under 18 years, diagnosis of terminal cancer, diagnosis of diseases, other than heart failure that could cause volume overload, such as end-stage renal disease requiring hemodialysis, severe liver disease, pericardial tamponade or constriction, acute myocardial infarction, and primary valvular disease, diagnosis of acute coronary syndrome, procedures that might affect prognosis (cardiac bypass surgery, coronary artery stenting), and lack of contact with the hospital. The diagnosis of ADHF was confirmed if the admitting cardiologist included heart failure as the primary admitting diagnosis and the documented physical examination laboratory and radiologic findings were consistent with this diagnosis. Following the index HF admission the medical record was assessed to determine hospital readmission, with a follow-up period of up to 44 months. Patients with a non-cardiovascular death prior to HF readmission were censored. Data was obtained from the comprehensive review of the medical record and cardiologist’s admission note regarding medical history and physical examination. The baseline characteristics of patients were collected upon hospital admission except the New York Heart functional class (NYHA) that was obtained from the most recent clinic visit when the patient was clinically stable prior to the index admission. When admitted, all patients were in NYHA class III or IV.

### Transthoracic echocardiography and global longitudinal strain

Standard 4-chamber, 3-chamber and 2-chamber apical views and parasternal short-axis views of the left ventricle were obtained using a commercially available ultrasound system. All images were stored digitally and analyzed with offline software (Syngo Dynamics 9.0 software, Siemens Medical Solutions). The majority of echocardiography studies were performed within 24 h after admission, with all studies being completed by 48 h post-admission. We used these images to calculate GLS and EF that were included in the analysis.

Speckle tracking for myocardial strain was performed using Velocity Vector Imaging software (Siemens Medical Systems, Erlangen, Germany). A digital loop was acquired from apical 2–3 and 4 chamber views. The GLS was the average result of three measurements for each view. The software calculated the endocardial average strain values from 6 left ventricle segments for a total of 18 segments, therefore the GLS was the result of the average of 18 segments. We obtained GLS only in the case of adequate tracking quality at least in 5 of the 6 segments per view. LV ejection fraction was averaged from the three apical views by automated endocardial tracking of end-diastolic and end-systolic volumes. All measurements were made blinded to other results and clinical details.

#### Adequacy of measurements

Multiple studies have demonstrated the low measurement variability for GLS [6–10]. In our software, left ventricle endocardial borders were manually traced at the end-diastole in ECG-gated long axis views. Subsequently, the software’s automatic border tracking algorithm, which tracks image features throughout the whole cardiac cycle, was applied. Accurate tracking was ascertained by visual assessment of all borders. Images with inadequate tracking of the endocardial were excluded from the analysis.

#### Outcome

Readmission to UI-Health for HF following the index ADHF admission was assessed using the electronic medical record.

#### Statistical analysis

The number of patients and percentages were calculated for categorical variables. Means, standard deviations, and medians were calculated for continuous variables. Receiver operating characteristic (ROC) curve was used to assess the optimal GLS threshold value which maximized the average of sensitivity and specificity for predicting HF admission. The area under the curve, which was a measure of the discriminatory power of the predictor, ranged from 0.5 to 1. Clinical characteristics were compared among patients categorized by quartiles of GLS, using the Chi-square test for categorical variables and *t*-test for continuous variables. Univariate and multivariate cox proportional hazard models were used to examine the association of GLS and HF readmission. Variables significantly correlated with outcomes on univariate analysis (*p* < 0.05) or known to influence outcomes were incorporated into the multivariate analyses. Accordingly, hazard ratios (HR) and 95% confidence interval (CI) were calculated. The Kaplan-Meier curves and log-rank tests were used to compare the time to first heart failure readmission across quartiles of GLS. Statistical analyses were conducted using IBM SPSS 21 (Armonk, NY). The significance level was set at 0.05.

## Results

### Population characteristics

In 204 (70%) patients, LV GLS by velocity vector imaging of the 2, 3 and 4-chamber views could be assessed. As shown in Table 1, mean age was 63.8 ± 15.2 years, 42% were male, and ethnicity distribution was 71% African Americans, 7% Caucasian and 7% Hispanics, the remaining 15% a mixture of other minorities. 49 patients had heart failure with preserved ejection fraction (HFpEF) (EF > 50%). The average LVEF was 40% and the average NYHA functional class during the clinic visit prior to their index hospitalization was 2.03. None of the patients was treated with coronary revascularization, implantable cardioverter defibrillator or cardiac resynchronization therapy after index hospitalization.

**Table 1:** Baseline characteristics of the entire cohort and by quartiles of GLS

Characteristic	Whole population	LV GLS				*P Value*
		< −14.15	–14.5 to −10.55	–10.54 to −6.41	> −6.41	
Age (years)	63.8 ± 15	66.3 ± 14	63.4 ± 15.8	65.7 ± 13.9	59.7 ± 16.3	0.116
Male	87 (42.4%)	11 (21.6%)	19 (37.3%)	25 (48.1%)	32 (62.7%)	<0.001
Sodium level	138.3 ± 3.4	138.1 ± 4.2	139 ± 3	138.7 ± 3.4	137.5 ± 2.9	0.153
Creatinine	2.2 ± 2.4	2.6 ± 3.6	2.6 ± 2.5	1.7 ± 1.2	1.8 ± 1.5	0.163
LVEF	40.4 ± 17.4	60.4 ± 7.5	47.8 ± 11.6	30.9 ± 10.4	22.3 ± 5.9	<0.001
Medication use						
ACEI or ARBs	140 (69.7%)	25 (49%)	32 (65.3%)	39 (78%)	44 (86.3%)	<0.001
β-Blocker	173 (86.1%)	37 (72.5%)	43 (87.8%)	47 (94%)	46 (90.2%)	0.011
Hydralazine	55 (27.5%	14 (27.5%)	21 (43.8%)	9 (18%)	11 (21.6%)	0.023
Ca channel Blockers	45 (22.6%)	17 (33.3%)	19 (39.6%)	6 (12%)	3 (6%)	<0.001
Loop diuretics	164 (81.6%)	41 (80.4%)	37 (75.5%)	41 (82%)	45 (88.2%)	0.43
Aldosterone-antagonists	19 (9.7%)	1 (2%)	3 (6.3%)	5 (10.4%)	10 (19.6%)	0.021
NYHA functional class						
Class I	55 (28.8%)	17 (36.2%)	16 (34%)	14 (28.6%)	8 (16.7%)	
Class II	78 (40.8%)	19 (40.4%)	15 (31.9%)	24 (49%)	20 (41.7%)	0.087
Class III	55 (28.8%)	9 (19.1%)	16 (34%)	10 (20.4%)	20 (41.7%)	
Class IV	3 (1.6%)	2 (4.3%)	0	1 (2%)	0	
Blood pressure (mmHg)						
Systolic	148.7 ± 38.4	158.5 ± 35.9	166.5 ± 38	145.2 ± 36.6	124.7 ± 29.7	<0.001
Diastolic	84.9 ± 21.6	83.1 ± 23.5	89.1 ± 22.2	86.1 ± 21.9	81.3 ± 18.2	0.28
Heart rate (bpm)	86.1 ± 21.5	79.2 ± 19.1	86 ± 20.3	87.7 ± 22.2	91.6 ± 23.1	0.032
History of ischemic heart disease	80 (39%)	13 (25.5%)	23 (45.1%)	27 (51.9%)	17 (33.3%)	0.029
Chronic obstructive pulmonary disease	30 (14.6%)	6 (11.8%)	10 (19.6%)	6 (11.5%)	8 (15.7%)	0.617
Diabetes	79 (38.5%)	15 (29.4%)	23 (45.1%)	24 (46.2%)	17 (33.3%)	0.206
Hypertension	190 (93.1%)	46 (90.2%)	50 (98%)	49 (94.2%)	45 (90%)	0.324

**Fig. 1:**
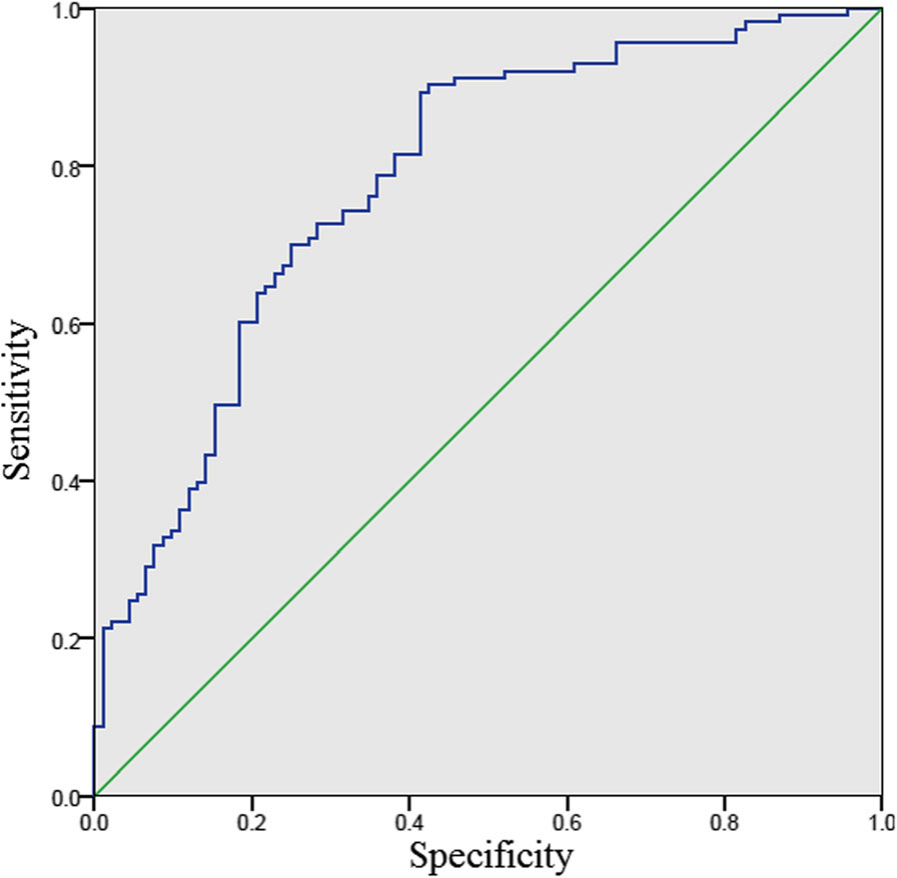
ROC curve. The area under the ROC curve for prediction of HF readmission using LV GLS was 0.783 (*p* < 0.001)

The area under the ROC curve for prediction of HF readmission using LV GLS was 0.783 (Fig. 1). Patients were grouped into quartiles according to LV GLS as follows, < −14.15%, −14.15% to −10.55%, −10.54% to −6.41% and > −6.41% demonstrating increased readmission with worse strain quartiles. Baseline characteristics of patients with a GLS above and below the threshold value are summarized in Table 1.

### Readmission

**Fig. 2:**
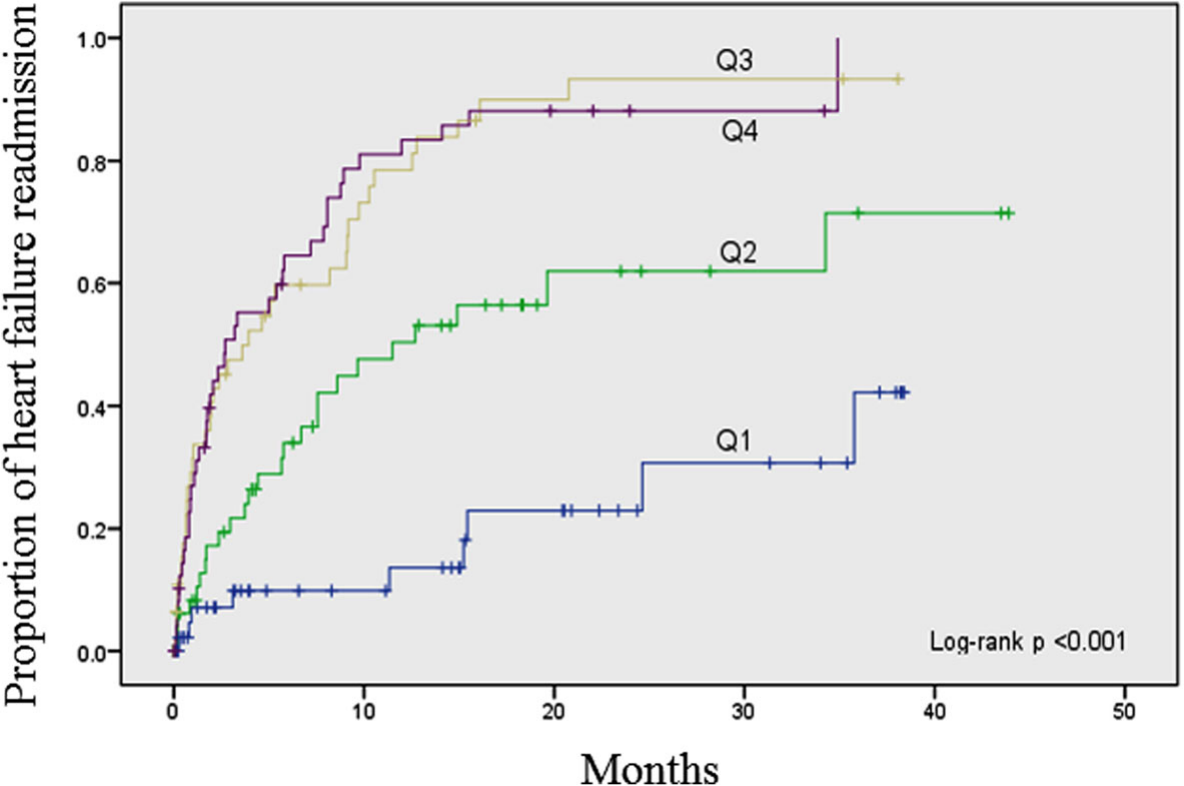
Kaplan-Meier curves. Kaplan-Meier curves showing higher heart failure readmissions in patients within worse LV GLS quartiles (Q). Q1 < −14.15, Q2 -14.15 to 10.55, Q3 -10.54 to −6.41, Q4 > −6.41

A total of 113 patients (55%) had at least one readmission. In the first quartile there were 10 (9%) patients with at least one readmission, 24 patients (21%) in the second quartile, 38 patients (34%) in the third quartile and 41 patients (36%) in the fourth quartile. Kaplan-Meier curves (Fig. 2) demonstrated significantly higher admission rate in the more altered GLS group (*p* < 0.001). On univariate Cox proportional hazard analyses (Table 2), more altered GLS was significantly associated with HF admissions, either as continuous variable (HR 1.17, CI 1.12-1.23, *p* <0.001) or with lower quartiles of GLS −14.15 to −10.55 (HR 3.1, CI 1.46-6.74, p 0.003), −10.54 and −6.41% (HR 7.4, CI 3.55-15.39, *p* <0.001), and > −6.41% (HR 7.8, CI 3.79-16.34, *p* <0.001). Additional variables significantly associated with HF admission in univariate analysis included LVEF (*p* <0.001), NYHA functional class III (p 0.01), systolic blood pressure (*p* < 0.001), diastolic blood pressure (*p* 0.025), history of ischemic heart disease (*p* 0.021), dementia (*p* 0.018) sodium level (*p* 0.018) and angiotensin converting enzyme inhibitors-angiotensin receptor blockers (ACEI-ARBs) use (*p* 0.008). In the multivariate analysis (Table 3), after adjusting for age, sex, history of ischemic heart disease, dementia, NYHA class, LV ejection fraction, use of ACEI or ARBs, systolic and diastolic blood pressure on admission and sodium level on admission, worse LV GLS was the strongest predictor of recurrent HF readmission either as continuous variable (HR 1.23, CI 1.09-1.4, *p* = 0.001) or using the cut-off point of −14.15 to −10.55 (HR 3.6, CI 1.26–7.9, p 0.014), between −10.54 and −6.41% (HR 5.19, CI 1.7-15.82, *p* <0.001), and > −6.41% (HR 5.3, CI 1.43–19.6, *p* <0.001). Ejection fraction was a univariate predictor for readmission, but not a multivariate predictor. The small number of patients with preserved LVEF precluded a useful analysis of this subgroup.

**Table 2:** Univariate analyses of factors that were shown to be significantly correlated to HF readmissions

HF Readmission			
Variable	Hazard ratio	95% CI	*p*-value
LV GLS –14.15 to –10.55	3.144	1.46–6.74	0.003
LV GLS –10.54 to 6.41	7.40	3.55–15.39	<0.001
LV GLS > −6.41	7.87	3.79–16.34	<0.001
LV GLS as a continuous variable ^a^	1.17	1.12–1.23	<0.001
LVEF	0.97	0.96–0.98	<0.001
Age	1.01	0.99–1.02	0.099
Male	1.11	0.77–1.61	0.566
NYHA functional class II	1.3	0.77–2.19	0.314
NYHA functional class III	2.01	1.18–3.41	0.01
NYHA functional class IV	0.92	0.12–6.87	0.937
Systolic blood pressure	0.98	0.98–0.99	<0.001
Diastolic blood pressure	0.99	0.98–0.99	0.025
Heart rate	1	0.99–1.01	0.137
Creatinine	0.9	0.82–1	0.052
Sodium level	0.93	0.88–0.98	0.018
History of ischemic heart disease	1.55	1.07–2.24	0.021
Diabetes	1	0.68–1.46	0.983
Hypertension	1.45	0.59–3.55	0.418
Dementia	12.18	1.54–95.95	0.018
Chronic obstructive pulmonary disease	1.24	0.76–2.01	0.382
ACEI-ARBs	1.83	1.17–2.86	0.008

^a^A different model with the same variables was performed using LV GLS as a continuous variable

**Table 3:** Multivariate Cox regression analysis incorporating factors that were shown to be significantly correlated with HF readmissions in univariate analyses

Variable	Number/Mean/Percentage	Hazard Ratio	95% Confidence Interval	*p*-value
LV GLS Quartile 2 (−14.15 to −10.55)	*N* = 51	3.16	1.26–7.90	0.014
LV GLS Quartile 3 (−10.54 to −6.41)	*N* = 52	5.19	1.70–15.82	0.004
LV GLS Quartile 4 (> − 6.41)	*N* = 51	5.30	1.43–19.60	0.012
LV GLS as a continuous variable^a^	−10.6 ± 4.7%	1.23	1.09–1.40	0.001
Age	63.8 ± 15	1.02	1.002–1.03	0.027
Left ventricular ejection fraction	40 ± 17%	0.98	0.95–1.02	0.288
History of ischemic heart disease	39%	1.13	0.71–1.78	0.608
NYHA III	29%	1.79	0.98–3.29	0.060
Use of ACEI/ARBs	70%	0.96	0.565–1.65	0.894
Systolic blood pressure	149 ± 38	0.999	0.99–1.01	0.689
Sodium level	138 ± 3	0.94	0.88–1.00	0.53

^a^A different model with the same variables was performed using LV GLS as a continuous variable

A dedicated statistical analysis to evaluate the correlation between GLS and all-cause readmission did not demonstrate a statistically significant correlation (data not shown).

## Discussion

In this study, we demonstrate that after adjusting for factors that can affect clinical outcomes, LV GLS is a strong and independent predictor of HF readmission following an index admission for ADHF. This is the first study to show GLS can predict readmission in a racially diverse group of patients with ADHF.

We know of only two other studies to assess strain in patients with ADHF. One of the previously published studies differed from our study in several important ways [11]. Whereas our study included patients with both heart failure with reduced ejection fraction (HFrEF) and HFpEF, the previous study appears to have focused on HFrEF. Furthermore, they found global circumferential strain (GCS) to predict outcome and not GLS. Our study found GLS to be an independent predictor. GLS is thought to be an early marker of subclinical LV dysfunction, whereas GCS becomes abnormal later in the course of myocardial disease [12]. It is unclear why they did not find GLS to be predictive, as one would expect this parameter to become abnormal before GCS. One might hypothesize that GLS would be a better predictor than GCS in a lower risk group due to its ability to detect myocardial dysfunction at an earlier stage.

The only other study to demonstrate GLS as a predictor of readmission [10] had a population consisting of 98% white Europeans and their follow-up was only 30 days. This differs from our study which included a largely African American patient population. This is an important discovery considering data exists to suggest African American patients with heart failure remodel differently than whites, raising the possibility that GLS would predict differently in this patient population [13].

Despite dramatic improvement in outcomes with medical therapy for heart failure, readmission rates remain high, with approximately 50% of HF patients rehospitalized within 6 months of discharge [14]. Although studies have found factors that can predict readmission in large administrative databases, assembling a risk model that can reliably predict readmission in individual patients has been less than successful [15, 16]. The ability to identify a group at high risk for readmission using GLS might allow for resources to be directed towards these individuals in order to reduce the readmission rate.

## Limitations

This was a single center study with information being obtained in a retrospective manner. The clinical use of GLS is limited considering its feasibility in only 70% of cases in our study. Also GLS evaluation by echocardiography requires good quality images in order to obtain reliable measurements. Two dimensional and Doppler echocardiographic parameters were not included in the present study.

## Conclusion

Our study is the first to demonstrate GLS as a predictor of readmission in a largely minority patient population with ADHF, which was independent of LVEF and other known clinical risk predictors. Further study to determine if GLS can impact outcomes in these patients is warranted.

## Abbreviations

ACEI: Angiotensin converting enzyme inhibitors
ADHF: Acute decompensated heart failure
ARBs: Angiotensin receptor blockers
CI: Confidence interval
GCS: Global circumferential strain
GLS: Global Longitudinal Strain
HF: Heart Failure
HFpEF: Heart failure with preserved ejection fraction
HFrEF: Heart failure with reduced ejection fraction
HR: Hazard ratio
LV: Left ventricle
LVEF: Left ventricular ejection fraction
NYHA: New York Heart Association
ROC: Receiver operating characteristic
UI-Health: University of Illinois Hospital and Health Sciences System
